# Could We Really Use* Aloe vera* Food Supplements to Treat Diabetes? Quality Control Issues

**DOI:** 10.1155/2017/4856412

**Published:** 2017-12-06

**Authors:** Solomon Habtemariam

**Affiliations:** Pharmacognosy Research Laboratories & Herbal Analysis Services UK, University of Greenwich, Medway Campus, Chatham Maritime, Kent ME4 4TB, UK

## Abstract

Diabetes UK has recently listed a number of herbs and spices that have been clinically shown to improve blood glucose control in type-2 diabetes patients and the diabetes high-risk group. With* Aloe vera *being top in this list, its health benefit along with health and beauty/food retailers supplying it was illustrated in detail. Previous article from this laboratory scrutinised the merit of using* A. vera* as an alternative therapy to prescription antidiabetic drugs and the risk of using food supplements in the market which do not qualify as drug preparations. In continuation of this discussion, the present study assesses three Aloe Pura brands and one Holland and Barret brand of* A. vera* juice supplements in the UK market through chromatographic and spectroscopic analysis. While the polysaccharide active ingredient, acemannan, appears to be within the recommended limit, it was found that Aloe Pura (one of the best-selling brands for* A. vera* supplement) products have benzoate additive that does not appear in the supplement levels. Moreover, two of the Aloe Pura brand juices contain methanol, suggesting that the International Aloe Science Council (IASC) certification does not guarantee the medicinal quality of these products. The therapeutic fitness of such supplements is discussed.

## 1. Introduction

Throughout the history of mankind, nature has provided unlimited access to potential medicines from the diverse flora, fauna, and mineral resources [1]. Perhaps our ancestors experimented by using the whole plant, whole roots, or leaves and then moved on to their extracts, often by using a blend of several plant parts or species, but the preferred preparation of drugs of natural origin in the modern era is based on purified single chemical entities [2]. Advances in drug discovery through chemical synthesis and technologies within the pharmaceutical/academic industries in the last few decades have also shifted the balance of drug development from natural to synthetic origin [3]. The large number of our prescription drugs to date tracing their origin back to natural resources [3–5], however, is a vivid reminder to the prominent role still played by natural products in our modern pharmacotherapies. Overall, while traditional medicine practices based on the whole plant or crude plant extracts remain part of the healthcare system in many developing countries, they are largely represented in Western countries to date by exceptionally few examples of prescription drugs [e.g., [6]]. On the other hand, there are a plethora of evidences to show the ever-increasing global impact of the food supplement industry. Some market research estimates the global dietary supplements business in 2016 as USD 132.8 billion, which is further expected to reach USD 220.3 billion by 2022 [7]. As dietary supplements are also often sold with various health claims, rigorous quality assurance measures to guarantee the products quality, safety, and efficacy must be employed to ensure consumer health protection.

**Figure 1:**
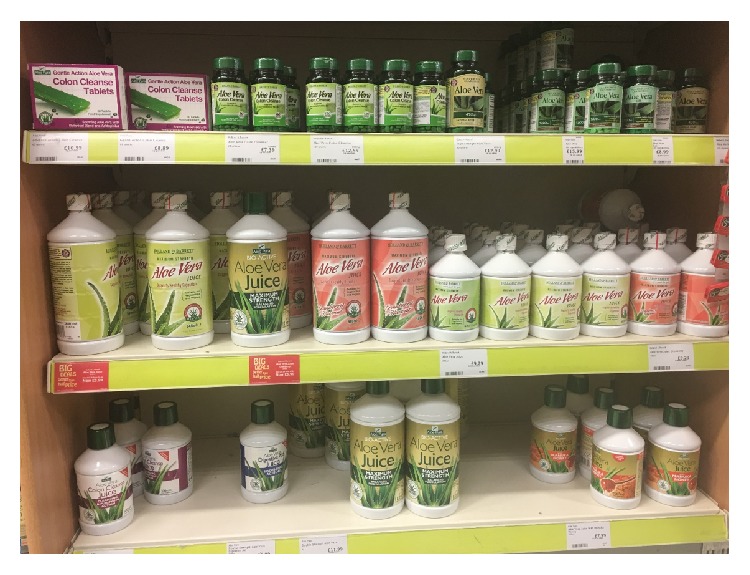
*Aloe vera* products available to the general public at the Holland and Barrett outlet.

In the previous report from this laboratory [8], the merit of using* Aloe vera* food supplements as antidiabetic drugs was scrutinised following the listing of the plant along with others by the Diabetes UK website [9] under the heading of “Herbal and Natural Therapies” [10]. It was highlighted that the recommendation by the Diabetes UK website for people to get potential therapy from the healthcare outlets like the “Holland and Barrett” could not be justified as there has been no consensus among the scientific community on using* A. vera* by its own as antidiabetic drug in the first place [8]. It was also highlighted that the various health supplements formulated for other diseases often with blends of other plants could not be justified as modern therapy for diabetes given the lack of scientific/clinical evidence for such formulations [8]. In this communication, the checks and balances of these formulations are further scrutinised through the use of an electronic nose, analytical instruments. For this purpose, four* Aloe vera* juices supplements were selected from the Holland and Barrett outlet (Figure 1): three of which were Aloe Pura brands bearing the International Aloe Science Council (IASC) certificate stamp, while one was that of Holland and Barrett's own brand (Table 1). In view of their composition, this article scrutinises the existing quality control measure of* A. vera* food supplements in their potential use as antidiabetic drug therapies. Data from chromatographic and spectroscopy analysis showing the inadequacy of the IASC certificate as a quality measure is discussed.

## 2. Materials and Methods

### 2.1. *Aloe vera* Juice Samples

Four* A. vera* juice samples were obtained from the shelves of Holland and Barret outlets (Figure 1). Their batch number and content with respect to* A. vera* and other ingredients mentioned therein as well as preservatives and antioxidants listed are also detailed (Table 1). The four samples were the subject of analysis and scrutiny as detailed in the following sections.

### 2.2. HPLC Analysis

An Agilent 1200 series gradient HPLC system composed of degasser (G1322A), quaternary pump (G1322A), autosampler (G1329A), thermostat column compartment (G1316A) maintained at 25°C, and a diode array detector (G1315D) was used. Samples were diluted in the buffer/solvent immediately before analysis and injected (20 *μ*L) onto a reverse phase column (Agilent, Eclipse Plus C18, 5 *μ*m, 4.9 × 150 mm). The mobile phase composition for routine analysis of components of diverse polarity range was made by using a mixture of water (A) and methanol (B). The composition of the mobile phase at a flow rate of 1 ml/min was rising from 10% to 90% B over a period of 50 minutes. For the preservatives analysis, an isocratic solvent system comprising a mixture of 5 mM ammonium acetic buffer of pH 4.4 and methanol in the ratio 25 : 75 (v/v) was used. The detection of the compounds was based on UV absorption as indicated in Results and Discussion.

### 2.3. NMR Analysis

The measurement of* A. vera *components by NMR has been carried out by following the American Herbal Pharmacopoeia guidelines [11]. For this, the JEOL 500 MHz instrument using D_2_O as a solvent containing 4,4-dimethyl-4-silapentane-1-sulfonic acid (DSS) reference was used.

### 2.4. Gas Chromatography Analysis

Following the detection of methanol in some of the* A. vera* samples by NMR, accurate quantification was done by using gas chromatography. Samples were analysed using an Agilent Technologies 6850 Network GC system (DB5 capillary column, 30 m, internal diameter of 0.25 mm, and 0.25 *μ*m film thickness), with a split/splitless injection system used in the split mode (1 : 50). Samples were delivered via the Agilent Technologies 7683B series injector system at injection temperature of 150°C, injection volume of 1 *μ*l, and FID detection made at 250°C. The GC oven temperature was programmed at isothermal condition at 30°C. Helium was used as the carrier gas at constant flow rate of 1 ml/min.

## 3. Results and Discussion

**Figure 2:**
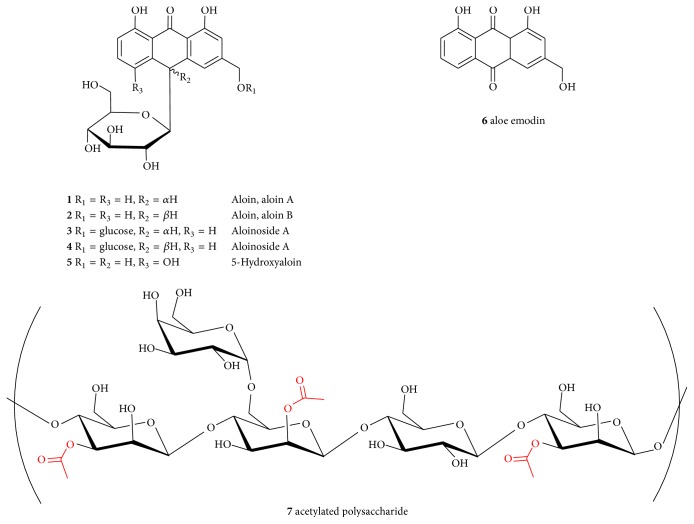
Structures of some of the key pharmacologically active compounds of* Aloe vera*.

Both the greenish leafy skin and inner white/colorless parenchymatous tissue (source of* Aloe* gel) contain a long list of bioactive compounds including amino acids, peptides, and proteins/enzymes, carbohydrates (unique mono-, di-, and polysaccharides), minerals and vitamins, and secondary metabolites belonging to the general class of anthraquinones/anthrones, chromones, phytosterols, and phenolics. Several review articles describing the composition and potential pharmacological/industrial application of* A. vera* are available [e.g., [12–16]]. Individually, a range of these compounds have shown some degree of antidiabetic activity by their own but the polysaccharides of* Aloe* gel have been mostly regarded as the main active component for antidiabetic effects, while anthraquinones/anthrones are attributed to the general purgative effect of* Aloe*  [15, 16]. The bitter anthraquinones-dominated exudate obtained from the outer skin of* Aloe* leaves (*Aloe* latex) is often used for beverages flavouring while their laxative effects associated with purgative activity are an interest in pharmaceutical preparations [17, 18]. The most important purgative principle of* A. vera* is aloin(a mixture of two isomers: aloin A** (1)** and B (also named barbaloin,** 2**)), although a number of similar compounds including dimeric forms and anthraquinones (primarily aloe emodin,** 6**) are also known to occur in the plant (Figure 2). The smooth muscle stimulant effect of such compounds (primarily by the major component, aloin) which is of interest in short-term weight loss formulations and/or constipation therapy (like that by Senokot/Senna) is a limitation in using* A. vera *for long-term therapy or as food supplements. Accordingly, aloin is listed among the substances which shall not be added to food (Regulation (EC) number 1334/2008), while the 88/388/EEC of 22 June 1988 directive [19] limits the use of aloin as 0.1 mg/kg in food and as high as 50 mg/kg in alcoholic beverages.* Aloe vera* juice products certified by the IASC should also limit aloin (A and B) in the finished products to 10 ppm or less. As the health benefits of* A. vera* supplements are claimed to be realized after long-term intake (about three months), methodologies for industrial scale removal of anthraquinones/anthrones (laxative components) are now routinely employed during the manufacturing process. Accordingly, all the four* A. vera* food supplement samples taken for the present analysis display “aloin removed” marking in their labels (Table 1).

**Figure 3:**
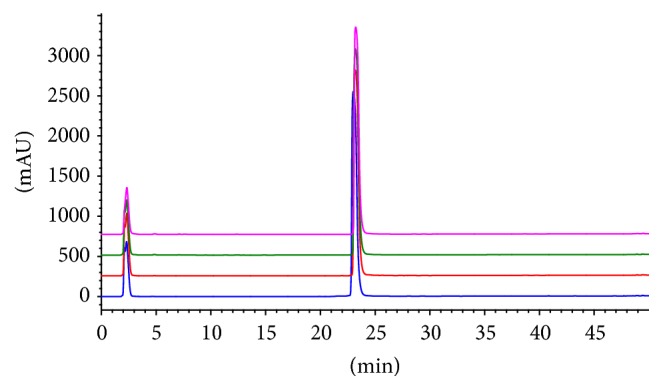
HPLC profile of* Aloe vera* juices. From lower to upper traces are AV-1, AV-2, AV-3, and AV-4, respectively. The chromatogram was obtained from 5-fold dilution of the samples and UV monitoring at 250 nm. Peaks at 2.1 and 23.2 min represent the ascorbic acid antioxidant and potassium sorbate/sodium benzoate preservative additives, respectively. The instrumental set-up was as described in Materials and Methods and the mobile phase was a mixture of water and methanol.

One of the most common methods of laxatives removal is through nonspecific decolourisation (aloin and other anthraquinones/anthrones** (1–6)** being orange/yellow in colour) by activated carbon or charcoal [11]. Various other methods of removal and* A. vera* preparations for food and medicine are also reported [20, 21]. The method of aloin removal from the Aloe juice food supplements in this study (Table 1) is, however, not known. Analysis of these samples by HPLC using UV detection at 250 nm confirmed the removal of these phenolic compounds: in fact, the two principal components of these products by the HPLC methodology of analysis were ascorbic acid and potassium sorbate/sodium benzoate which are the antioxidant and preservative additives, respectively (Figure 3). Similar results were obtained when the chromatogram was monitored at 280 and even 215 nm (data not shown) where the weakest chromophores are usually picked up. It is worth noting that the chromatographic system employed using a methanol gradient from 10 to 90% was optimised to detect a range of secondary metabolites from glycosides to nonpolar constituents. Hence, despite the fact that phenolic compounds in medicinal plants are known to be attributed to antioxidant and antidiabetic mechanisms of action [22–30], the modern* A. vera* medicine/food supplement preparations appear to have lost these groups of natural products. For example, anthraquinones of* A. vera* such as aloe emodin and their glycosides have been shown to have antidiabetic effect both* in vitro* and* in vivo *[31–35]. They (including aloin) also display anti-inflammatory effects both in cellular and animal models [36, 37]. Similarly, the therapeutic potentials of aloesin and aloe chromones have been well recognised [38, 39]. All these phenolic natural products are missing in* Aloe* food supplements as they are effectively removed by the industrial processing procedures employed. Other studies on* A. vera* food supplements also reported that polyphenols including flavonoids and tannins, which are effectively removed during the industrial processing, cannot be detected [40].

Despite the fact that two of the Aloe Pura products (AV-1 and AV-2) are labelled to contain a blend of formulations with a range of other plant products (Table 1), the HPLC profile (Figure 3) does not appear to show phenolic compounds in any comparable concentrations to the additives. As it is intriguing to find such high level of antioxidant/preservatives as prominent peaks of the HPLC analysis and the Aloe Pura products are being sold as 100% natural juice, it was worthwhile to quantify this additive by using authentic compounds as external standard. By constructing a concentration-response curve for ascorbic acid which gives straight line equations with *r*^2^ value of 0.999, its concentration in the* A. vera* juices could be quantified. As shown in Table 2, all of the products have around 1.5 mg/ml of ascorbic acid (~0.15% of the products (w/v)) and, as explained in the following text, this is equivalent to the active ingredient that is claimed to be responsible for the potential antidiabetic effect.

**Figure 4:**
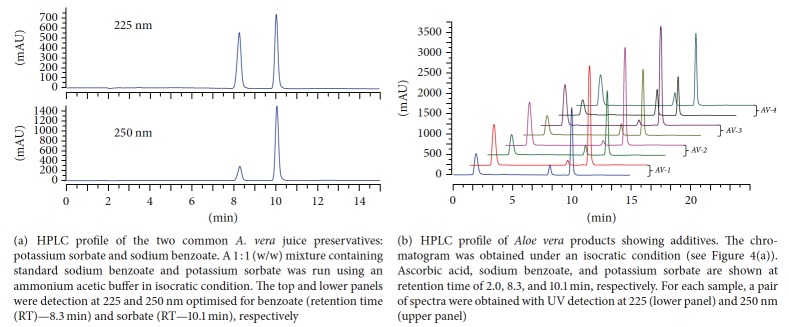


The chromatographic peak at 23.2 min (Figure 3) which corresponds to potassium sorbate is very intriguing. First, AV-4 which is labelled to contain sodium benzoate preservative in addition to potassium sorbate did not give additional peak, while quantitative measure using potassium sorbate standard did not give the expected concentration-dependent correlation graph with wider range of linearity. It was thus suspected that potassium sorbate and sodium benzoate are coeluted under the chromatographic system leading to the appearance of the peak at 23.2 min at even much higher concentration than expected. Analysis of the UV spectrum of this peak at 23.2 min and spiking studies confirmed the two compounds being coeluted together (data not shown). Hence, a chromatographic method that allows the resolution of the two preservative drugs was developed. As shown in Figure 4(a), the use of ammonium acetate buffer (0.39 g/L (5 mM), pH = 4.4, by using glacial acetic acid) and methanol in the ratio of 25 : 75 (v/v) as an isocratic elution system resulted in the complete separation of the two preservatives. On this basis, the detection and quantification of these preservatives in all* A. vera* samples were established. As shown in Figure 4(b), Aloe Pura products (AV-1, AV-2, and AV-3) which bear labels to contain only potassium sorbate as preservative contain benzoate additive in good amounts. Quantitative analysis of these preservatives using this methodology is shown in Table 2. As explained in the latter section, the presence of benzoate in all of the samples analysed was further confirmed by NMR.

Fresh* A. vera* gel is about 99% water and must contain the polysaccharide active component, principally acemannan (Figure 2). The presence of acetylated polysaccharides at or above 5% dry weight in* A. vera* leaf juice and inner leaf juice has been recommended by the IASC [11] but variability from 1.2 to 10.2% has been reported in the various literature. The best methodology for the detection and quantification of* Aloe* polysaccharide is the ^1^H NMR spectroscopy and it was employed herein to analyse the four juice products in question following the standard procedure published by the American Pharmacopoeia [11].

**Figure 5:**
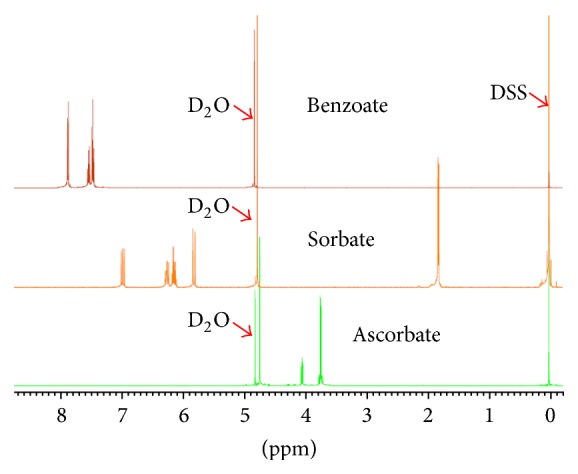
^1^H NMR spectra of standard additives of* Aloe vera*. The overlapped spectra of ascorbic acid, potassium sorbate, and sodium benzoate standards are shown. Calibration of the chemical shift was made by setting the DSS reference at zero ppm.

**Figure 6:**
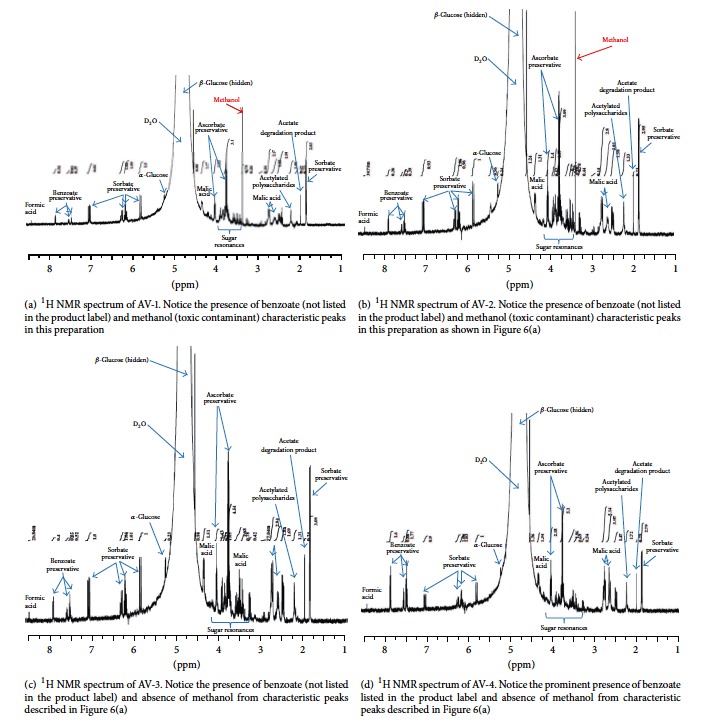


The ^1^H NMR profile of the three common additives of* A. vera* products is shown in Figure 5. On this basis, the three Aloe Pura products that are supposed to contain only potassium sorbate as preservative clearly have benzoate as evidenced from the characteristic peaks at *δ* 7.87 (2H), 7.55 (1H), and 7.49 (2H) (Figure 6). Since these characteristic peaks were evident in AV-3 which does not have the blended formulation with other herbal products, while they prominently occur in AV-4 which is stated in its label to have this preservative, the detection of benzoate additive in Aloe Pura products (in good agreement with the HPLC results) could be confirmed by NMR. On the basis of the ^1^H NMR integral analysis, the ratio of the sorbate to benzoate seems to be ~4 : 1 in Aloe Pura products, while estimation of their concentration using the more accurate quantitative HPLC method is presented in Table 2. Thus, the labelling of the three Aloe Pura brand products along with the “100% stabilized* Aloe vera* juice” claim needs a revision.

The acetylated polysaccharides markers at ~*δ* 2.1 in the ^1^H NMR spectrum appear to have very small integral value as compared to the additives and the calculated estimated value using the established procedures [11, 41, 42] was less than 0.2% (Table 2). Considering that* Aloe vera* juice is ~99% water, there is no doubt that these products meet the IASC requirement of ≥5% acetylated polysaccharides on dry weight basis. It was noticed, however, that the acetylated polysaccharide(s) ^1^H NMR signal appear to diminish over time even when the products were kept in the fridge following the opening of the bottle (data not shown). The variability of* A. vera* products in acemannan content depending on various manufacturing conditions such as drying has also been described in the various literature [e.g., [43]]. Hence, despite the presence of the above-said stabilisers (antioxidant and preservatives), the quality of* A. vera* juice can considerably change over time (within a week) and the big bottle (0.5–1 L) products for the stated small daily doses (Table 1) do not seem to be a reasonable packaging methodology. Fresh* A. vera* gel that we obtain from home-grown material also appears to lose considerable amount of acetylated polysaccharide(s) when the juice was treated with charcoal and filtration (data not shown). Hence, keeping the active components of* A. vera* juice intact is indeed a challenging task that could also be attributed to the numerous variability of pharmacological data reported from animal and clinical studies [8]. Variability in the acylated polysaccharide content with respect to biological activity has been reported. For example, complete deacetylation not only alter the chemical/physical properties of* A. vera* acemannan's but also their biological effects [44].

**Figure 7:**
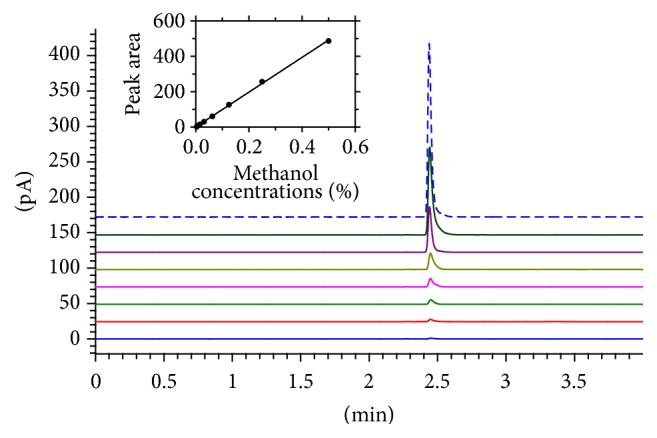
Standard GC chromatograms of methanol. From top to bottom are 0.5, 0.25, 0.125, 0.0625, 0.03125, 0.015625, 0.007813, and 0.003906% methanol concentrations. The inset shows the calibration curve constructed from the data.

The ^1^H NMR has also been acclaimed for detecting unwanted contaminants or adulterants in* A. vera *products [11, 41]. Interestingly, two of the Aloe Pura products which are blended with a number of other plant extracts in the formulation (AV-1 and AV-2) appear to contain methanol as evidenced from the characteristic peak at ~*δ* 3.36 (Figures 6(a) and 6(b)). No food or medicinal product should contain this toxic chemical and the detection of methanol as a significant peak in the ^1^H NMR spectrum is worrying. By employing a more accurate GC instrumentation, the concentration of methanol in these products was quantified. For this, a methanol reference extremal standard (Figure 7) was used to plot a calibration graph with *r*^2^ value of 0.999. Accordingly, the presence of methanol in the two samples (AV-1 and AV-2) which was detected by the ^1^H NMR was quantified at ~0.02% (Table 2). Although this concentration of methanol for the small volume of daily doses recommended by the product manufacturers (Table 1) may not cause visible toxicity, the presence of such toxic contaminant is highly undesirable.

The IASC certificate issued on the Aloe Pura products (except for the incomplete preservative labelling and methanol contaminant) and quality assurance guarantee in Holland and Barrett juices appear to be the beginning of good standard practices within the industry. These checks and balances, however, were far too inadequate as methanol was already detected in the IASC certified Aloe Pura products which also contained the preservative benzoate that does not appear in the labels. Some of the products (AV-1 and AV-2) also claimed to have a blend of many botanical additives (Table 1), although it is hard to detect (let alone quantify) them by the ^1^H NMR and HPLC analysis employed herein. While botanical blends are used for specific benefits of the gastrointestinal system, their relevance to diabetes needs other sets of scientific evidence.

In conclusion, medicinal plants with potential to treat diseases including diabetes could be accepted as a therapeutic option if the formulations are presented with proof of efficacy and stringent measures to ascertain their quality and safety.* Aloe vera* products containing the IASC or equivalent certificate of assurance could be a good starting point for standardising the products and optimising them for research/clinical trials on their antidiabetic potential. As a drug preparation, however, the existing quality assurance system for these food supplements does not appear to be sufficient even to guarantee what the label says. In the absence of unequivocal clinical evidence available for such formulations and quality assurance measures that mirror pharmaceutical products, these products cannot be recommended for treating diabetes.

## Figures and Tables

**Table 1:** Description of selected *A. vera* juices scrutinized in this analysis.

*Aloe vera* juice products	Brand, code	Description
Colon cleanse juice, cleansing and purifying action	Aloe Pura, *AV-1*	*Batch number: 6194-1* (i) Whole leaf and unfiltered *A. vera* inner gel—100% stabilised *A. vera* juice (ii) Aloin removed (iii) *A. vera* whole leaf and inner gel (iv) Botanical blend *(Glycyrrhiza glabra)* [liquorice], *Taraxacum officinale* [dandelion], L-glutamine, flaxseed fibre [from *Linum usitatissimum*], apple pectin (from *Malus domestica*) 0.3%, and fructooligosaccharides *Additives:* antioxidant (ascorbic acid), preservatives (potassium sorbate). *Directions:* take 25 ml once or twice daily

Digestive aid juice, soothing action	Aloe Pura, *AV-2*	*Batch number: 6290-1* (i) Whole leaf Aloe Vera and inner gel, unfiltered—100% stabilised *A. vera* juice (ii) Aloin removed (iii) Botanical blend (*Mentha piperita* [peppermint], *Carica papaya* [papaya], *Chamomilla recutita* [chamomile], *Foeniculum vulgare* [fennel], bromelain from *Annas comosus*). *Additives:* antioxidant (ascorbic acid), preservatives (potassium sorbate). *Directions:* take 25 ml once or twice daily

BIO-ACTIVE *Aloe vera* juice, maximum strength	Aloe Pura, *AV-3*	*Batch number: 6349-1* (i) Whole leaf *A. vera* and inner gel, unfiltered—100% stabilised *A. vera* juice (ii) Aloin removed (iii) Natural *A. vera* juice (99.99%) *Additives:* antioxidant (ascorbic acid), preservatives (potassium sorbate). *Directions:* no dose recommendation

Maximum strength *Aloe vera* juice supports healthy digestion	Holland and Barrett, *AV-4*	*Batch number: 6332-4* (i) *A. vera* leaf juice 99% with antioxidant (ascorbic acid) and preservatives (ii) A combination of filtered whole leaf and unfiltered inner gel *A. vera* (iii) Aloin removed *Additives:* antioxidant (ascorbic acid), citric acid, and preservatives (potassium sorbate, sodium benzoate). *Directions:* take one tablespoon (15 ml) one to three times daily

**Table 2:** Components of juice products. Symbols indicate the following: ^*∗*^calculated by GC and ^*∗∗*^calculated by HPLC; mean and SEM values are shown. ^*∗∗∗*^Representative result from average of two classical experiments based on estimation by ^1^H NMR as described previously [11].

*Aloe Vera* juices	Methanol ^*∗*^ (%)	Ascorbic acid (mg/ml)	Potassium sorbate (mg/ml) ^*∗∗*^	Sodium benzoate (mg/ml)	Acylated polysaccharide (% w/w in juices) ^*∗∗∗*^
AV-1	0.01906 ± 0.002182	1.596 ± 0.08356	0.2903 ± 0.001489	0.05166 ± 0.0003262	0.0876
AV-2	0.01599 ± 0.0009745	1.534 ± 0.08485	0.2747 ± 0.0005745	0.05112 ± 0.0002120	0.1285
AV-3	ND	1.517 ± 0.04910	0.2808 ± 0.0006749	0.05805 ± 0.000017	0.1925
AV-4	ND	1.229 ± 0.01660	0.1610 ± 0.0003571	0.1341 ± 0.0001400	0.089

## References

[1] Chin Y., Balunas M. J., Chai H. B., Kinghorn A. D. (2006). Drug discovery from natural sources. *The AAPS Journal*.

[2] Habtemariam S. (2017). Going back to the good old days: The merit of crude plant drug mixtures in the 21st century. *International Journal of Complementary & Alternative Medicine*.

[3] Katiyar C., Gupta A., Kanjilal S., Katiyar S. (2012). Drug discovery from plant sources: an integrated approach. *AYU*.

[4] Chen S.-L., Yu H., Luo H.-M., Wu Q., Li C.-F., Steinmetz A. (2016). Conservation and sustainable use of medicinal plants: Problems, progress, and prospects. *Chinese Medicine*.

[5] Siddiqui A. A., Iram F., Siddiqui S., Sahu K. (2014). Role of natural products in drug discovery process. *International Journal of Drug Development and Research*.

[6] Ekor M. (2013). The growing use of herbal medicines: Issues relating to adverse reactions and challenges in monitoring safety. *Frontiers in Neurology*.

[7] Zion Market research, Dietary Supplements Market by Ingredients (Botanicals, Vitamins, Minerals, Amino Acids, Enzymes) for Additional Supplements, Medicinal Supplements and Sports Nutrition Applications—Global Industry Perspective, Comprehensive Analysis and Forecast, 2016–2022

[8] Habtemariam S. (2017). Could We Really Use Aloe Vera Food Supplements to Treat Diabetes?. *International Journal of Diabetes and Clinical Research*.

[9] Diabetes UK, “Herbal and Natural Therapies,” http://www.diabetes.co.uk/Diabetes-herbal.html (Accessed, 08 September 2017)

[10] Diabetes UK, “Aloe Vera and Diabetes,” http://www.diabetes.co.uk/natural-therapies/aloe-vera.html (Accessed, 08 September 2017)

[11] American Herbal Pharmacopoeia, *Aloe Vera* Leaf *Aloe Vera* Leaf Juice *Aloe Vera* Inner Leaf Juice *Aloe vera* (L.) Burm. f. Standards of identity, analysis, and quality control.” http://www.cosmesi.it/Portals/7/Documenti/Monograph%20AHP_Aloe_vera_leaf_.pdf

[12] Hamman J. H. (2008). Composition and applications of Aloe vera leaf gel. *Molecules*.

[13] Sahu P. K., Giri D. D., Singh R. (2013). Therapeutic and medicinal uses of *Aloe vera*: A review. *Pharmacology & Pharmacy*.

[14] Sánchez-Machado D. I., López-Cervantes J., Sendón R., Sanches-Silva A. (2017). Aloe vera: Ancient knowledge with new frontiers. *Trends in Food Science & Technology*.

[15] Baruah A., Bordoloi M., Baruah H. P. D. (2016). Aloe vera: a multipurpose industrial crop. *Industrial Crops and Products*.

[16] Radha M. H., Laxmipriya N. P. (2015). Evaluation of biological properties and clinical effectiveness of *Aloe vera*: a systematic review. *Journal of Traditional and Complementary Medicine*.

[17] Cirillo C., Capasso R. (2015). Constipation and botanical medicines: An overview. *Phytotherapy Research*.

[18] Sánchez-Machado D. I., López-Cervantes J., Mariscal-Domínguez M. F. (2017). An HPLC Procedure for the Quantification of Aloin in Latex and Gel from Aloe barbadensis Leaves. *Journal of Chromatographic Science (JCS)*.

[19] European Union, “European Council Directive 88/388/EEC of 22 June1988, http://eur-lex.europa.eu/legal-content/EN/ALL/?uri=CELEX%3A31988L0388(Accessed, 08 September 2017)

[20] The International Aloe Science Council, “Processing methods for *Aloe vera* leaf,” http://www.iasc.org/Portals/19/Documents/Scientific/16_0531_Decolorization_statement_final.pdf?ver=2016-05-31-163902-973 (Accessed, 08 September 2017)

[21] Ahlawat K. S., Khatkar B. S. (2011). Processing, food applications and safety of aloe vera products: a review. *Journal of Food Science and Technology*.

[22] Belwal T., Nabavi S., Nabavi S., Habtemariam S. (2017). Dietary anthocyanins and insulin resistance: When food becomes a medicine. *Nutrients*.

[23] Habtemariam S., Varghese G. K. (2014). The antidiabetic therapeutic potential of dietary polyphenols. *Current Pharmaceutical Biotechnology*.

[24] Habtemariam S. (2015). Investigation into the antioxidant and antidiabetic potential of moringa stenopetala: identification of the active principles. *Natural Product Communications (NPC)*.

[25] Roselli M., Lentini G., Habtemariam S. (2012). Phytochemical, antioxidant and anti-*α*-glucosidase activity evaluations of Bergenia cordifolia. *Phytotherapy Research*.

[26] Tsala D. E., Lannang A. M., Dimo T. (2016). Antidiabetic and wound healing effects of smeathxanthone a. *Recent Advances in Biology and Medicine*.

[27] Nabavi S. F., Habtemariam S., Daglia M., Shafighi N., Barber A. J., Nabavi S. M. (2015). Anthocyanins as a potential therapy for diabetic retinopathy. *Current Medicinal Chemistry*.

[28] Habtemariam S., Cowley R. A. (2012). Antioxidant and anti-*α*-glucosidase compounds from the rhizome of peltiphyllum peltatum (Torr.) Engl. *Phytotherapy Research*.

[29] Habtemariam S. (2011). *α*-Glucosidase inhibitory activity of kaempferol-3-O-rutinoside. *Natural Product Communications (NPC)*.

[30] Habtemariam S., Lentini G. (2015). The therapeutic potential of rutin for diabetes: an update. *Mini-Reviews in Medicinal Chemistry*.

[31] Pérez Y. Y., Jiménez-Ferrer E., Zamilpa A. (2007). Effect of a polyphenol-rich extract from *Aloe vera* gel on experimentally induced insulin resistance in mice. *American Journal of Chinese Medicine*.

[32] Li J., Ding L., Song B. (2016). Emodin improves lipid and glucose metabolism in high fat diet-induced obese mice through regulating SREBP pathway. *European Journal of Pharmacology*.

[33] Anand S., Saravanababu C., Lakshmi B. S., Muthusamy V. S. (2016). Aloe-emodin glycosides ameliorate glucose utilization via insulin downstream regulators: an in vivo investigation. *Asian Journal of Pharmaceutical and Clinical Research*.

[34] Anand S., Muthusamy V. S., Sujatha S. (2010). *Aloe emodin* glycosides stimulates glucose transport and glycogen storage through PI3K dependent mechanism in L6 myotubes and inhibits adipocyte differentiation in 3T3L1 adipocytes. *FEBS Letters*.

[35] Xue J., Ding W., Liu Y. (2010). Anti-diabetic effects of emodin involved in the activation of PPAR*γ* on high-fat diet-fed and low dose of streptozotocin-induced diabetic mice. *Fitoterapia*.

[36] Park M.-Y., Kwon H.-J., Sung M.-K. (2009). Evaluation of aloin and aloe-emodin as anti-inflammatory agents in aloe by using murine macrophages. *Bioscience, Biotechnology, and Biochemistry*.

[37] Park M.-Y., Kwon H.-J., Sung M.-K. (2011). Dietary aloin, aloesin, or aloe-gel exerts anti-inflammatory activity in a rat colitis model. *Life Sciences*.

[38] Yimam M., Brownell L., Jia Q. (2015). Aloesin as a medical food ingredient for systemic oxidative stress of diabetes. *World Journal of Diabetes*.

[39] Yimam M., Zhao J., Corneliusen B., Pantier M., Brownell L. A., Jia Q. (2013). UP780, A chromone-enriched aloe composition improves insulin sensitivity. *Metabolic Syndrome and Related Disorders*.

[40] López Z., Núñez-Jinez G., Avalos-Navarro G. (2017). Antioxidant and cytotoxicological effects of Aloe vera food supplements. *Journal of Food Quality*.

[41] Jiao P., Jia Q., Randel G., Diehl B., Weaver S., Milligan G. (2010). Quantitative ^1^H-NMR spectrometry method for quality control of aloe vera products. *Journal of AOAC International*.

[42] Campestrini L. H., Silveira J. L. M., Duarte M. E. R., Koop H. S., Noseda M. D. (2013). NMR and rheological study of Aloe barbadensis partially acetylated glucomannan. *Carbohydrate Polymers*.

[43] Minjares-Fuentes R., Rodríguez-González V. M., González-Laredo R. F., Eim V., González-Centeno M. R., Femenia A. (2017). Effect of different drying procedures on the bioactive polysaccharide acemannan from Aloe vera (Aloe barbadensis Miller). *Carbohydrate Polymers*.

[44] Chokboribal J., Tachaboonyakiat W., Sangvanich P., Ruangpornvisuti V., Jettanacheawchankit S., Thunyakitpisal P. (2015). Deacetylation affects the physical properties and bioactivity of acemannan, an extracted polysaccharide from Aloe vera. *Carbohydrate Polymers*.

